# Invited review: Frontotemporal dementia caused by *microtubule-associated protein tau* gene (MAPT) mutations: a chameleon for neuropathology and neuroimaging

**DOI:** 10.1111/nan.12213

**Published:** 2015-01-29

**Authors:** B Ghetti, A L Oblak, B F Boeve, K A Johnson, B C Dickerson, M Goedert

**Affiliations:** * Department of Pathology and Laboratory Medicine, Indiana University School of MedicineIndianapolis, USA; † Department of Neurology, Mayo ClinicRochester, USA; ‡ Department of Radiology, Massachusetts General Hospital and Harvard Medical SchoolBoston, USA; § Department of Neurology, Massachusetts General Hospital and Harvard Medical SchoolBoston, USA; ¶ Medical Research Council, Laboratory of Molecular BiologyCambridge, UK

**Keywords:** FTDP-17 *MAPT*, tau aggregation, neurofibrillary tangle, Pick body, tau, [F18]-T807

## Abstract

Hereditary frontotemporal dementia associated with mutations in the *microtubule-associated protein tau* gene (*MAPT*) is a protean disorder. Three neuropathologic subtypes can be recognized, based on the presence of inclusions made of tau isoforms with three and four repeats, predominantly three repeats and mostly four repeats. This is relevant for establishing a correlation between structural magnetic resonance imaging and positron emission tomography using tracers specific for aggregated tau. Longitudinal studies will be essential to determine the evolution of anatomical alterations from the asymptomatic stage to the various phases of disease following the onset of symptoms.

## Introduction

Inherited forms of frontotemporal dementia (FTD) have been known for many years [1–4], but as the clinical and pathological features are heterogeneous, the nomenclature has been variable, with disorders being called familial Pick disease, familial progressive subcortical gliosis, familial presenile dementia with tangles, autosomal-dominant parkinsonism and dementia with pallido-ponto-nigral degeneration. The major clinical manifestations include behavioural disturbances, aphasia, cognitive impairment and parkinsonism. Individuals from 13 families, with FTD and genetic linkage to chromosome 17q[21–22], were presented at a Consensus Conference at the University of Michigan in 1996 [5]. It was agreed that the unifying name should take into account the clinical features, as well as the genetic linkage, rather than the neuropathology, which was incomplete. Tau inclusions had been described in affected individuals from only four of the 13 families. Thus, the concept of FTD and Parkinsonism linked to Chromosome 17 (FTDP-17) was born. The disorder in one family had been named ‘multiple system tauopathy with presenile dementia’ (MSTD) [6]. As a result, the term ‘tauopathy’ was also introduced, and it is often used to refer to disorders in which tau protein deposition is the predominant feature.

In June 1998, mutations in the *microtubule-associated protein tau* gene (*MAPT*) were reported in affected individuals from nine of the 13 families [7–9]. They all suffered from a dementia syndrome, whereas some also had parkinsonism. The central neuropathologic feature was the presence of filamentous hyperphosphorylated tau protein in neurons or in both neurons and glia. The remaining four families had mutations in the *Granulin* gene (*GRN*), which is 1.54 megabase pairs centromeric to *MAPT* [10],[11]. Thus, FTDP-17 has been divided into FTDP-17 *MAPT* and FTDP-17 *GRN* [12].

FTD associated with *MAPT* mutations is a disorder that affects multiple domains including behaviour, language, memory and motor function. It often begins with psychiatric symptoms and can mimic Pick disease, primary progressive aphasia, Alzheimer disease (AD), progressive supranuclear palsy (PSP) or corticobasal degeneration (CBD). Neuropathology and neuroimaging reveal diverse pictures, consistent with variability of the clinical phenotype. It is important for clinicians, neuropathologists and imaging researchers to be aware that *MAPT* mutations can cause such a protean disorder. Their discovery established that tau dysfunction alone can cause neurodegeneration of multiple neuronal systems and dementia.

## Epidemiology

To date, 53 pathogenic *MAPT* mutations have been reported in approximately 150 families [13] from Asia, Australia, Europe, and both North and South America. Molecular genetic analyses have demonstrated that some families share a common founder [14].

FTDP-17 *MAPT* affects men and women equally. The average age at symptom onset is 49 years, with a range from the early 20s to late 70s, similar to sporadic frontotemporal lobar degeneration (FTLD). The average life expectancy after symptom onset is 8.5 years, with a range from 1.5 to 26 years [15–17].

Disease phenotypes in patients with the same *MAPT* mutation may vary significantly within and between families, as well as between individuals with different mutations [16],[18],[19]. Thus, genetic modifiers and/or environmental factors may underlie the phenotypic variability in clinical presentation.

## Genetics and molecular pathology

FTDP-17 *MAPT* is inherited in an autosomal-dominant manner. The *MAPT* gene, located on chromosome 17q21, encodes the tau protein, which was discovered in 1975 [20]. A decade later, the intraneuronal inclusions of AD and Pick disease were found to be immunoreactive for hyperphosphorylated tau [21–23]. The neurofibrillary tangles (NFTs) of AD are composed of paired helical and straight filaments. Their molecular characterization established that they are made of tau protein [24–26].

**Figure 1:**
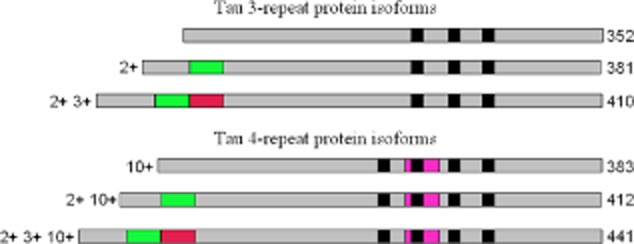
Schematic representation of the six tau isoforms generated by alternative mRNA splicing of exons 2, 3 and 10.

In the adult human brain, six tau isoforms are generated from *MAPT*, the tau gene, through alternative mRNA splicing (Figure 1) [27]. Alternative splicing of exon 10 gives rise to three isoforms with three microtubule-binding repeats (3R) each and three isoforms with four microtubule-binding repeats (4R) each. The repeats are 31 or 32 amino acids in length and are located towards the carboxy-terminus. In addition, the presence of inserts of 29 or 58 amino acids or no insert in the amino-terminus gives rise to 1 N, 2 N or 0 N forms of each 3R and 4R tau. Full-length tau assembles through the repeats that form the core of paired helical and straight filaments. In developing human brain, 3R tau predominates, while in adult brain, the concentrations of 3R and 4R tau are approximately equivalent. A normal ratio of wild-type 3R to 4R tau appears to be essential for preventing neurodegeneration and dementia in the human brain in mid-life.

Between 1994 and 1997, familial forms of FTD were linked to chromosome 17q[21–22], a region that contains *MAPT* [28–30]. In parallel, neuropathological and biochemical studies showed abundant tau deposits in neurons and glia [31–34]. They highlighted the presence of a tauopathy affecting grey and white matter in the absence of amyloid beta deposition, and directed several laboratories towards the search for mutations in *MAPT*. In 1998, the first mutations were reported in exons 9, 10 and 13, as well as in the splice site of intron 10 [7–9]. The vast majority of known mutations occurring in the coding region are in the repeats, with the mutant tau proteins having a reduced ability to interact with microtubules [35–37].

Exonic mutations are missense, silent or deletion. All but two (R5H and R5L in exon 1) occur in exons [9–13]. Most intronic mutations are clustered in the 5′-splice site of the intron following exon 10. These intronic mutations and some exonic mutations located in exon 10 affect the alternative mRNA splicing of exon 10, causing a relative increase of 4R tau [8],[9],[38],[39]. They destabilize a stem-loop structure at the exon 10 5′-splice site intron junction or disrupt *cis*-acting elements in exon 10. Existence of a stem-loop structure was hypothesized [8],[9] at the time of the discovery of mutations in *MAPT*, in view of the self-complementarity of this region, with subsequent work supporting this hypothesis [40–42]. The determination of the solution structure of an oligonucleotide corresponding to the exon/intron junction refined the stem-loop model, with the identification of an adenosine bulge between the sixth and seventh base pairs [43]. Mutations S305I, S305N, S305S, +3, +4, +11, +12, +13, +14 and +16 destabilize the stem-loop, resulting in increased U1 snRNP binding, and enhanced exon 10 inclusion. Mutations in exon 10, located outside the stem-loop, can also increase exon 10 splicing, because of the strengthening of exon splicing enhancers or the weakening of exon splicing silencers [39],[44].

Thus, the primary effect of the coding region mutations may be equivalent to a partial loss of function. The net effect of mutations, whose primary effect is at the RNA level, is the overproduction of wild-type 4R tau, which interacts more strongly with microtubules than 3R tau [45]. Some mutations, such as P301L, P301S and P301T in exon 10, affect only [20–25]% of tau molecules, with [75–80]% being wild-type, arguing against a simple loss of function mechanism as an important disease determinant.

It is therefore possible that a partial loss of function of tau is necessary for setting in motion the gain of toxic function mechanism that will lead to neurodegeneration. For *MAPT* mutations with a primary effect at the RNA level, the overproduction of 4R tau may result in an excess of tau over available binding sites on microtubules, leading to the cytoplasmic accumulation of unbound 4R tau. This would probably require the existence of different binding sites on microtubules for 3R and 4R tau. Validation of this hypothesis will require structural information at the atomic level. An imbalance in isoform ratios could also affect tau aggregation directly. Studies *in vitro* have shown that filament assembly is decreased in reactions containing 3R and 4R tau when compared with those containing only 4R tau [46].

**Figure 2:**
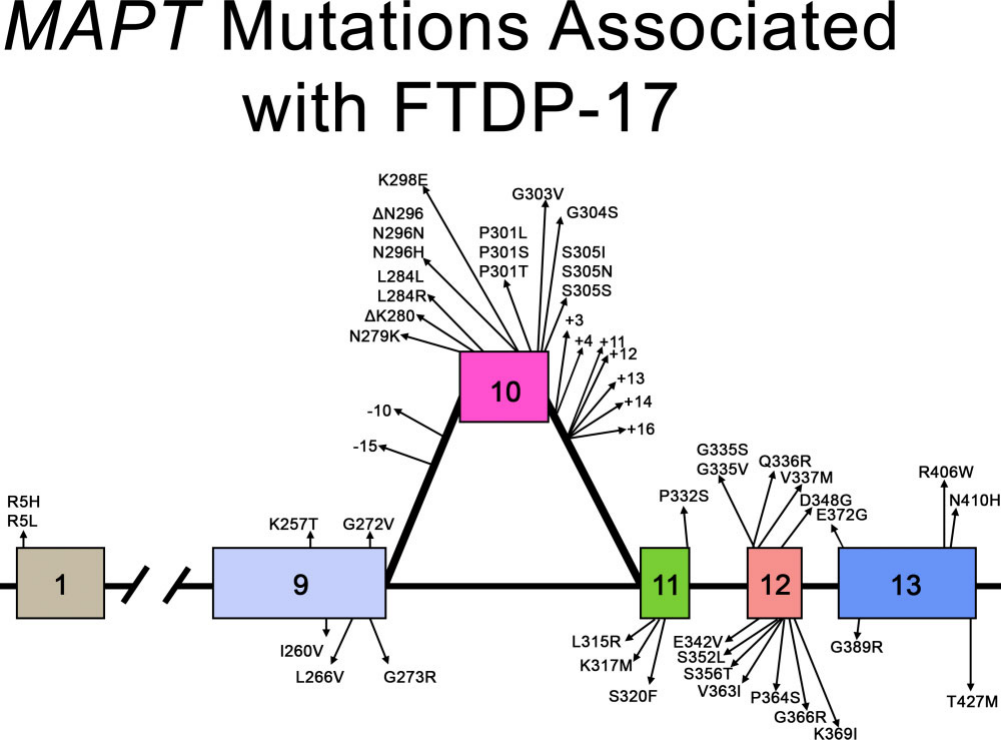
Schematic representation of the exons and introns of the *MAPT* gene, where 53 mutations causing FTDP-17 have been found. Intronic mutations −15 and +4 occur together.

Figure 2 shows the 53 mutations that are currently known[6–9,14,16,33,34,38,14,39,47–132]. The most common are N279K, P301L and intron 10^+16^.

### Soluble and insoluble Tau

A central question revolves around the process by which tau filaments form. In FTDP-17 *MAPT*, tau protein isoforms have biochemical characteristics that differ from those of the normal protein [133]. A mutation may result in a structurally abnormal protein, an abnormal ratio of 3R to 4R tau, or both. Normally, tau is a soluble protein; however, in FTDP-17 *MAPT*, it is found in both soluble and insoluble forms. Tau accumulates in the cytoplasm and becomes hyperphosphorylated, insoluble and assembles into filaments. However, the order of events in relation to hyperphosphorylation and filament formation is not clearly understood.

Missense mutations in exons 1, 9, 11, 12 and 13 affect all six tau isoforms. Missense and deletion mutations in exon 10 affect the alternative mRNA splicing of exon 10, altering isoform ratios in such a way that relatively more 4R than 3R tau is produced. A summary is given in Table 1, row 1.

**Table 1:** Western blot analysis, filaments and cellular inclusions associated with of *MAPT* mutations

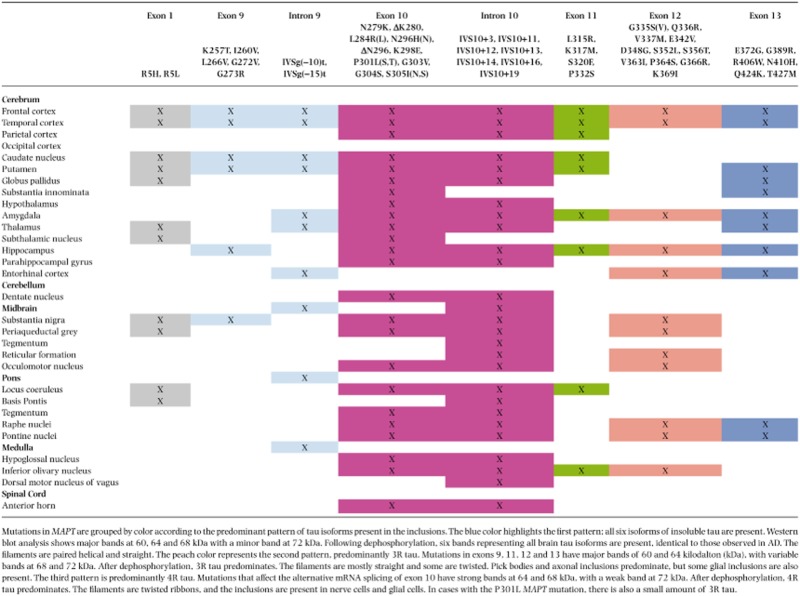

### Hyperphosphorylation of Tau and filament formation

Hyperphosphorylation of tau is believed to play a crucial part in the pathogenesis of human tauopathies [133]. In FTDP-17 *MAPT*, it is unlikely to be primary as none of the known mutations influence phosphorylation directly. Nevertheless, evidence has been adduced to suggest that some *MAPT* mutations can lead to enhanced phosphorylation [134], followed by filament formation. Morphological evidence for the presence of the insoluble form is provided by the finding that some tau deposits are fluorescent using Thioflavin S, tau filaments are found in neurons and glia and tau filaments can be visualized in sarkosyl-insoluble tissue preparations.

Filament morphologies have been studied using fixed tissues and preparations of dispersed filaments [135]. The latter are particularly informative as they allow one to correlate Western blot analysis with immunoelectron microscopy. Tau filaments can be straight, ribbon-like or paired helical. Table 1 summarizes the characteristics of abnormal tau as demonstrated by Western blot analysis, the type of tau filament and the nature of the intracytoplasmic inclusions.

## Distribution of Tau inclusions

The neuropathological phenotypes associated with FTDP-17 *MAPT* vary substantially; however, the invariable hallmark is the presence of tau protein deposits in neurons or in both neurons and glia. No cases with only glial tau inclusions have been described. Tau deposits are abundant in cerebral cortex and white matter; subcortical and brain stem nuclei, as well as the spinal cord, are variably affected.

Inclusions are labelled by antibodies specific for the amino-terminus, the repeat region and the carboxy-terminus of tau. In addition, phosphorylation-dependent antibodies are used. According to the numbering of the longest human brain tau isoform, prominent phosphorylation sites are serines 202, 214, 235, 262, 356, 396, 404 and 422, and threonines 181, 205, 212 and 231. An antibody recognizing phosphorylated S262 and/or S356 labels NFTs, but not classical Pick bodies [136]. Antibody AT8, which recognizes tau phosphorylated at S202 and T205, labels tau deposits in neurons and glia. Some tau deposits are also immunoreactive for ubiquitin. RD3 and RD4 are anti-tau antibodies that recognize 3R and 4R tau, respectively.

**Figure 3:**
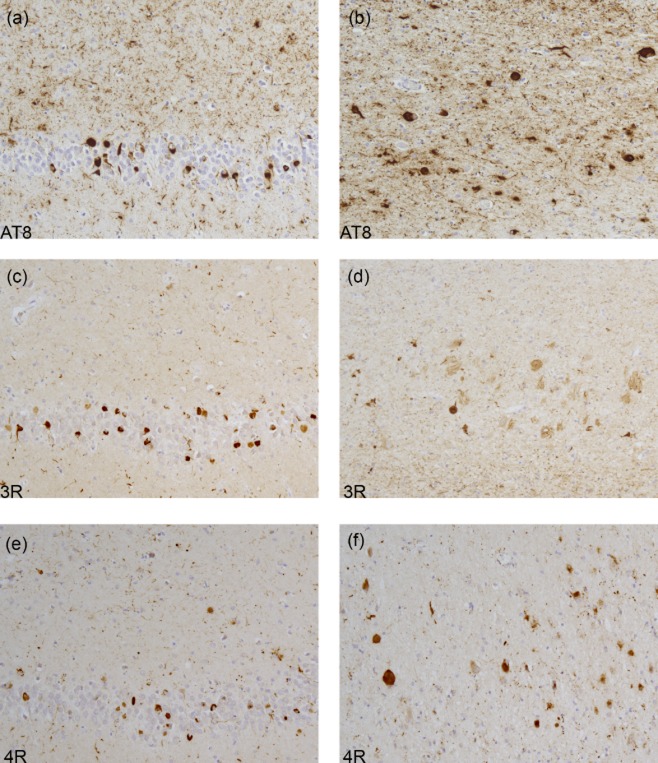
Tau pathology in the hippocampus of a patient carrying the R406W mutation. Dentate gyrus (a, c, e) and pyramidal layers (b, d, f) of the hippocampus are immunolabeled with anti-tau antibodies, showing tau-immunoreactive neuropil threads and neurofibrillary tangles with antibodies AT8 (a, b), 3R tau (c, d) and 4R tau (e, f).

**Figure 4:**
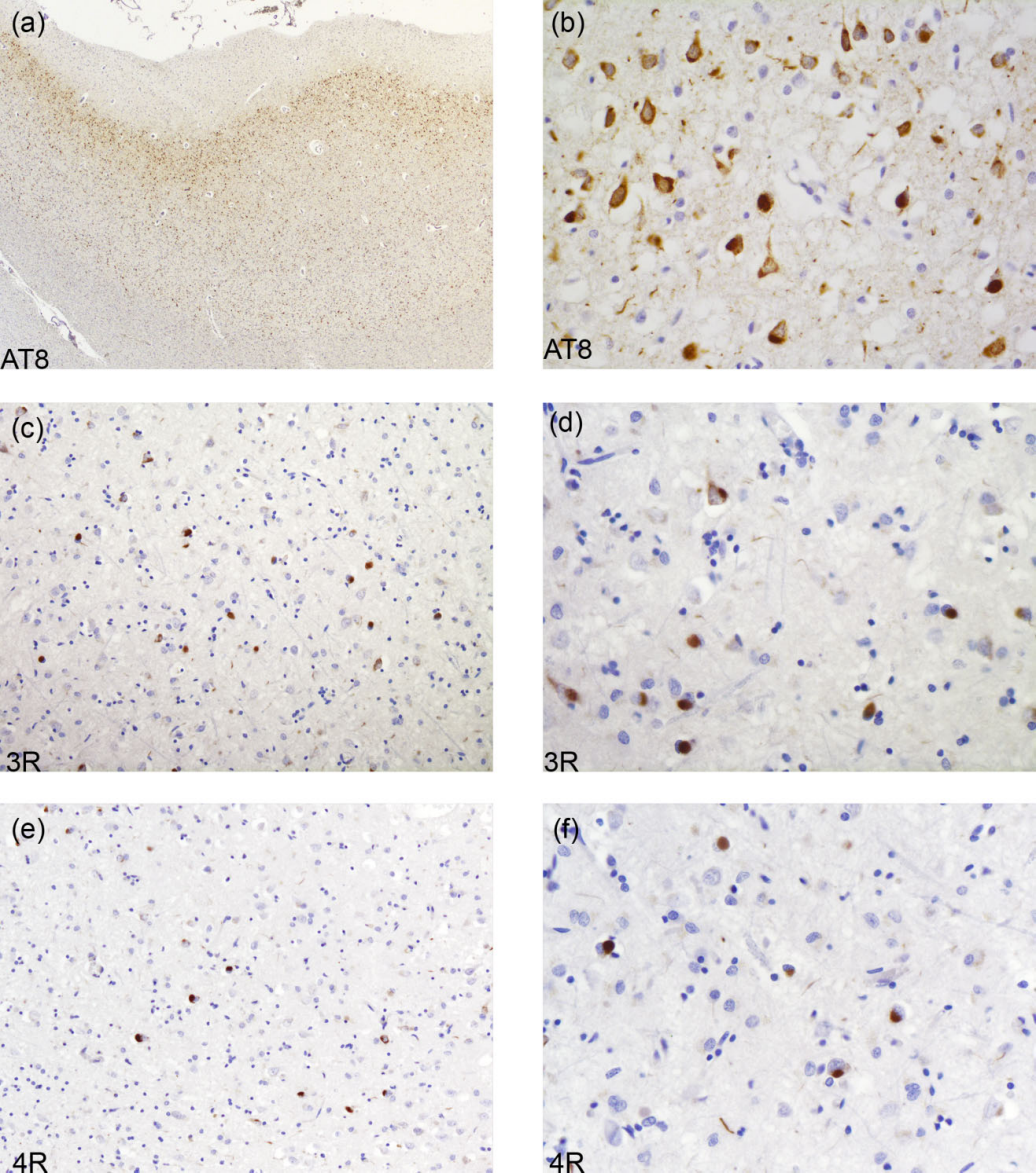
Tau immunohistochemistry in the frontal cortex from a case with the G389R mutation. AT8 labelling demonstrates tau-immunoreactive deposits or Pick bodies in neurons of layers II-VI (a, b). The tau deposits are positive for 3R (c, d) and 4R (e, f) tau.

**Figure 5:**
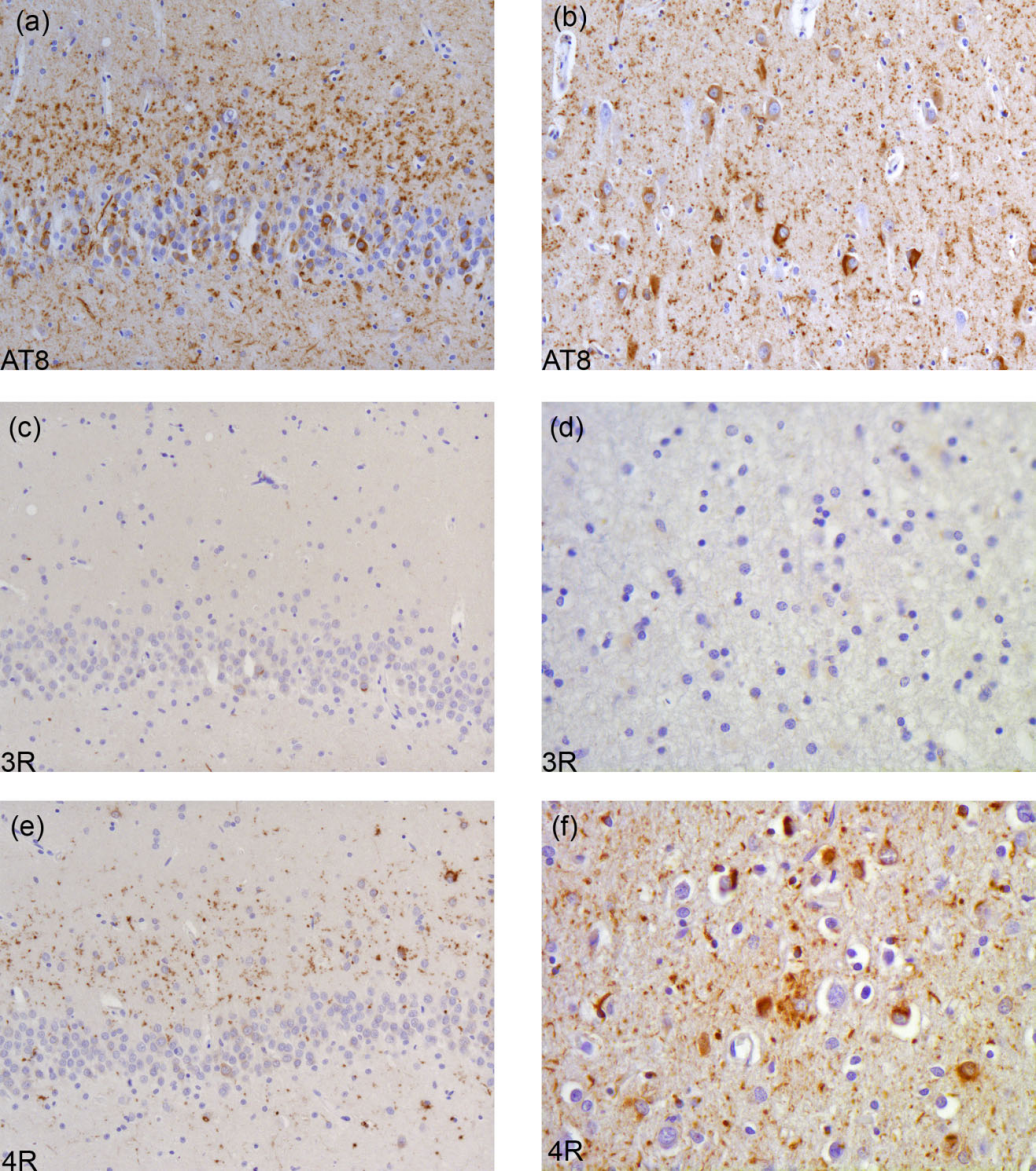
Tau pathology in the hippocampus of a patient carrying the IVS10+16 mutation. Dentate gyrus (a, c, e) and pyramidal layers (b, d, f) of the hippocampus are immunolabeled with anti-tau antibodies, showing tau-immunoreactive inclusions with antibodies AT8 (a, b) and 4R tau (e, f), but not 3R tau (c, d).

**Figure 6:**
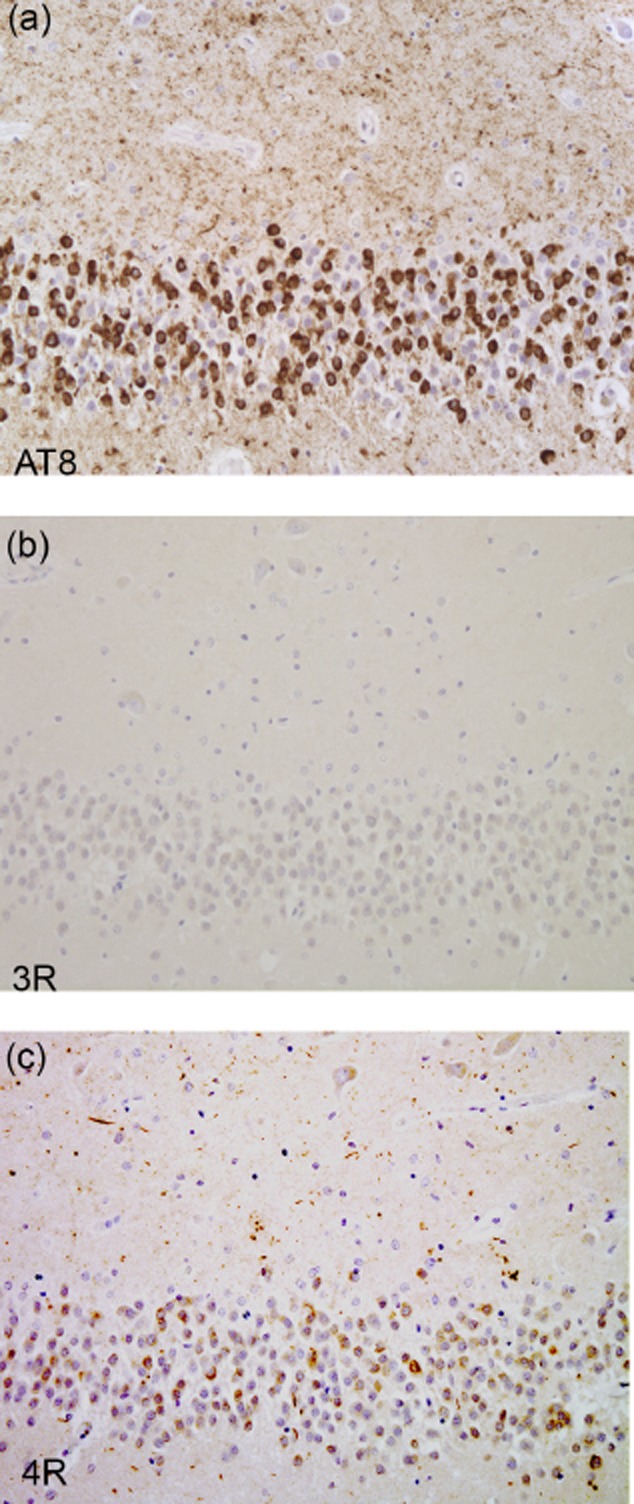
Tau pathology in the hippocampus of a patient carrying the P301L mutation. The dentate gyrus of the hippocampus is labeled with AT8 (a) and 4R tau (c), but not 3R tau (b).

Inclusions may resemble those of AD with filaments made of all six brain tau isoforms (see Table 1). This is the case of mutations V337M (Exon 12) and R406W (Exon 13), as illustrated in Figure 3. The images highlight neuronal involvement with tau immunopositivity revealed by an antibody specific for phosphorylated tau (AT8, Figure 3**a**,**b**), as well as by antibodies specific for 3R and 4R tau (Figure 3**c–f**). Inclusions similar to Pick bodies are often observed in association with mutations in exons 9, 11, 12 and 13. Straight filaments, with some twisted filaments, are characteristic of Pick body-like structures that are primarily composed of 3R tau, with a variable amount of 4R tau (Figure 4, Table 1). The images highlight Pick body-like inclusions immunopositive for phosphorylated tau (Figure 4**a**,**b**) and 3R tau (Figure 4**c**,**d**). There is occasional immunopositivity for 4R tau (Figure 4**e**,**f**). Mutations in exons 9, 11, 12 and 13 lead to deposits of tau filaments predominantly in neurons, while mutations in exons 1 and 10, as well as those in the introns following exons 9 and 10, are associated with neuronal and glial deposits. The glial pathology is in the form of coiled bodies in oligodendroglia, tufted astrocytes and astrocytic plaques, reminiscent of that of PSP and CBD. Cytoplasmic tau deposits affect the perikarya and dendrites of nerve cells. There is strong and diffuse cytoplasmic immunopositivity, but in Thioflavin S preparations, fluorescence is barely detectable, unlike what is seen in NFTs and Pick body-like inclusions. Twisted ribbon filaments characterize the neuronal and glial inclusions and are composed of 4R tau. Highlighted in Figure 5 are affected nerve cells and glial cells using antibodies specific for phosphorylated tau (Figure 5**a**,**b**) and 4R tau (Figure 5**e**,**f**). 3R tau staining was not observed (Figure 5**c**,**d**). Mutations in exon 10 only affect 4R tau; some of these mutations also affect exon 10 splicing, altering the ratio of 3R/4R tau. This is illustrated in Figure 6, where the immunohistochemical characteristics of neuronal and glial involvement in the hippocampus are revealed by antibodies specific for phosphorylated (**a**), 3R (**b**) and 4R (**c**) tau.

The anatomical distribution of tau in the various regions of the central nervous system has been reported with different details in relation to individual mutations. In Table 2, the brain regions involved in FTDP-17 *MAPT* are presented according to the mutation and grey matter regions involved.

**Table 2:** Brain areas affected in FTDP-17 *MAPT* according to mutation

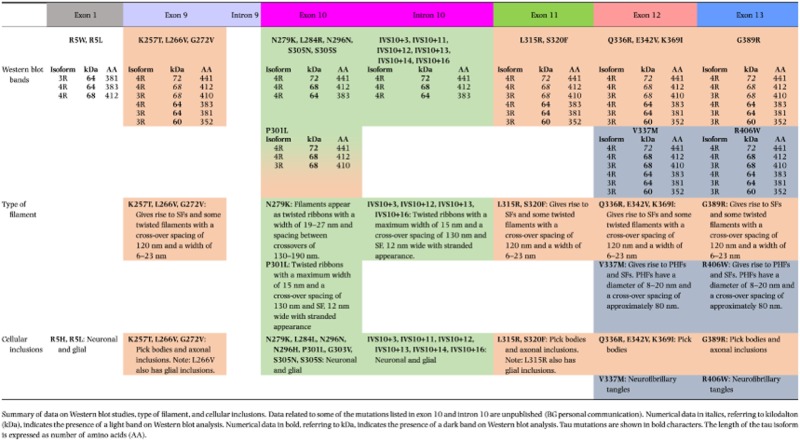

The data related to anatomical distribution are mostly obtained in intermediate and late stage of FTDP-17 *MAPT*. The degree of atrophy varies, with brain weights ranging from 654 to 1290 g. Little is known about the early neuropathologic stages. In the intermediate stages, atrophy of the cerebral hemispheres is mild, even though the characteristic histopathological changes in cerebral cortex, subcortical nuclei and white matter are already prominent. There may be mild atrophy of the caudate nucleus and a reduction in the pigmentation of the substantia nigra and the locus coeruleus. In advanced stages, the degree of atrophy varies and may be present throughout the frontal and temporal lobes, caudate nucleus, putamen, globus pallidus, amygdala, hippocampus and hypothalamus. Most often, the superior, middle and inferior frontal gyri, as well as the superior, middle and inferior temporal gyri, bear the brunt of the disease, with the anterior portion of the temporal lobe being particularly vulnerable. Brain atrophy may involve the frontal and temporal lobes asymmetrically and can be so severe that the gyri have a ‘knife edge’ appearance. The orbital, cingulate and parahippocampal gyri may also be involved. Parietal and occipital lobes are less frequently affected. The white matter of the centrum semiovale and the temporal lobes are often substantially reduced, as is the thickness of the corpus callosum. Midbrain and pons may also be reduced in bulk with particular involvement of the descending fibers of the fronto-pontine and temporo-pontine pathways. In addition, there is a reduction in the nigro-striatal projections. In some instances, mild atrophy of the cerebellar cortex and discoloration and atrophy of the dentate nucleus are present. The lateral ventricles and the third ventricle are enlarged.

## Neuroimaging

Computerized tomography (CT) and magnetic resonance imaging (MRI) of patients with *MAPT* mutations reveal atrophy of the frontal and/or temporal lobes with occasional involvement of the parietal lobes, accompanied by enlargement of the lateral ventricles [16,^74^,^82^,^96^,^121^,^137^,138]. In some individuals, the cortical atrophy is asymmetrical, but the majority of cases have relatively symmetric patterns of atrophy. MRI T2*-weighted images may show accumulation of paramagnetic substances (iron) in mesencephalic nuclei [137]. Increased T2-weighted signal changes have been reported [139]; they are often seen in white matter, reflecting the prominent white matter pathology present in many cases. It is not yet clear if these changes are due to a loss of myelinated axons; additional radio-pathological studies are needed.

A few studies on familial FTD have begun to compare neuroimaging features resulting from mutations in different genes. *MAPT* mutations are associated with a relatively symmetric atrophy of the anterior temporal lobe, accompanied by lesser atrophy of orbitofrontal and lateral prefrontal cortices. Preliminary findings indicate that *MAPT* mutations affecting the splicing of exon 10 are predominantly associated with medial temporal lobe involvement, while mutations in the coding region are mainly associated with lateral temporal lobe involvement. This is important because it begins to differentiate patients with *MAPT* mutations from those with *GRN* or *C9ORF72* mutations. *GRN* mutations tend to be associated with markedly asymmetric atrophy of the temporal, inferior frontal and inferior parietal lobes [138,^140^,141]. In contrast, *C9ORF72* mutations tend to be associated with symmetric atrophy predominantly involving dorsolateral, medial and orbitofrontal lobes, with additional loss in anterior temporal, parietal and occipital lobes, as well as in the cerebellum [141].

**Figure 7:**
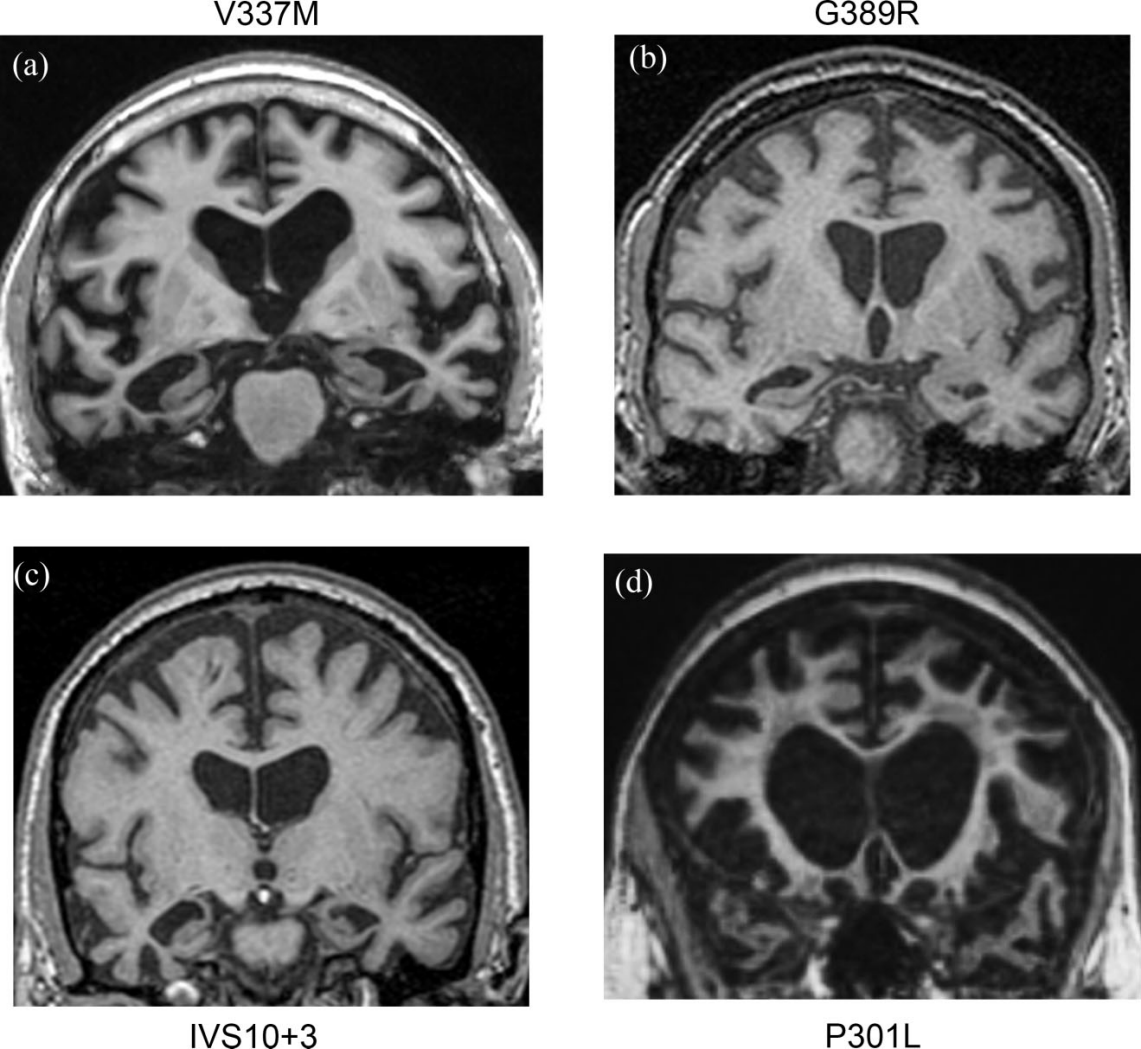
Coronal T1-weighted magnetic resonance imaging (MRI). Panel a is from a 65-year-old male with behavioural-variant frontotemporal dementia associated with the V337M *MAPT* mutation. Symptoms evolved over 20 years. Note the moderate to marked bilateral frontal and temporal cortical atrophy, with a severe anterior temporal lobe atrophy. Panel b is from a 25-year-old male with frontotemporal dementia and primary progressive aphasia associated with the G389R *MAPT* mutation. Symptoms rapidly developed over 1 year. Note the mild bilateral frontal and temporal cortical atrophy, with more pronounced medial and inferolateral anterior temporal atrophy. Panel c is from a 51-year-old male with behavioural-variant frontotemporal dementia associated with the IVS10+3 *MAPT* mutation. Symptoms evolved over 3 years. Note the mild bilateral frontal and temporal cortical atrophy with more pronounced mesial temporal atrophy. Panel d is from a 62-year-old female with severe behavioural-variant frontotemporal dementia associated with the P301L *MAPT* mutation. Symptoms evolved over 6 years. Note the striking bilateral prefrontal and anterior temporal atrophy with white matter changes.

An attempt to correlate structural brain imaging with the biological aspects of hereditary tauopathies may not be successful because of the different rates of atrophy and the sequences of anatomical involvement, which are highly variable even in cases with the same mutation. Figure 7 shows structural MRIs from patients who are carriers of *MAPT* mutations, V337M, G389R, IVS10+3 and P301L, which are respectively associated with inclusions containing 3R and 4R tau, predominantly 3R tau, predominantly 4R tau, and 4R with some 3R tau. An important confound in these comparisons is that these images are from individuals at different stages of disease, and specific details about the initial location of atrophy are no longer discernible.

Longitudinal MRI studies of brain atrophy suggest that *MAPT* mutations are associated with an atrophy rate intermediate between those of *GRN* and *C9ORF72* [142,143].

Functional imaging studies, such as single photon emission CT (SPECT) and [F-18] fluorodeoxyglucose positron emissions tomography (FDG-PET), typically demonstrate substantial abnormalities. FDG-PET often shows reduced frontal and/or temporal uptake, similar to the patterns seen in sporadic FTD [144]. PET with dopaminergic (e.g. [F-18]-fluoro-L-dopa (6FD) and [C-11]-raclopride) tracers reveals uptake abnormalities different from those of Parkinson disease (PD) [145]. In the MSTD family, a study of multiple members carrying mutation IVS10+3 showed that structural changes, predominantly seen bilaterally in the medial temporal lobes, substantially overlapped with the hypometabolism observed with FDG-PET [146].

Investigations have begun to determine whether neuroimaging abnormalities are present in asymptomatic *MAPT* mutation carriers, with initial evidence suggesting that abnormalities of brain structure [147], connectivity [148],[149] and white matter tract integrity [148] may be detectable prior to the development of symptoms. Longitudinal changes in an asymptomatic MSTD mutation carrier showed that whole brain volume (WBV) changes were −0.47%/year in the first 2 years of assessment and −1.83%/year in the following 5 years, indicating an acceleration of the rate of brain atrophy and suggesting the approaching threshold of a clinically recognizable symptomatology [150]. In five symptomatic MSTD patients, the average WBV changes were −2.47%/year. Findings from the Genetic FTD Initiative suggest that structural changes can occur 25 years prior to symptom onset in the hippocampus, 15 years in the amygdala, 10 years in the temporal lobe and 5 years in insula and cingulate [151].

PET ligands to study tau pathology *in vivo* have been developed [152–157]. A series of compounds was tested for selectivity of binding to tau pathology in post-mortem brain tissue from patients with AD pathology [158]. Binding was compared against immunohistochemistry, and based on more than 25-fold greater binding to tissue sections with high tau burden relative to amyloid-β, [F-18]-T807 was selected; the first set of images and quantitative binding data of [F-18]-T807 to specific brain regions in a small group of patients with AD and normal controls was very encouraging [159].

**Figure 8:**
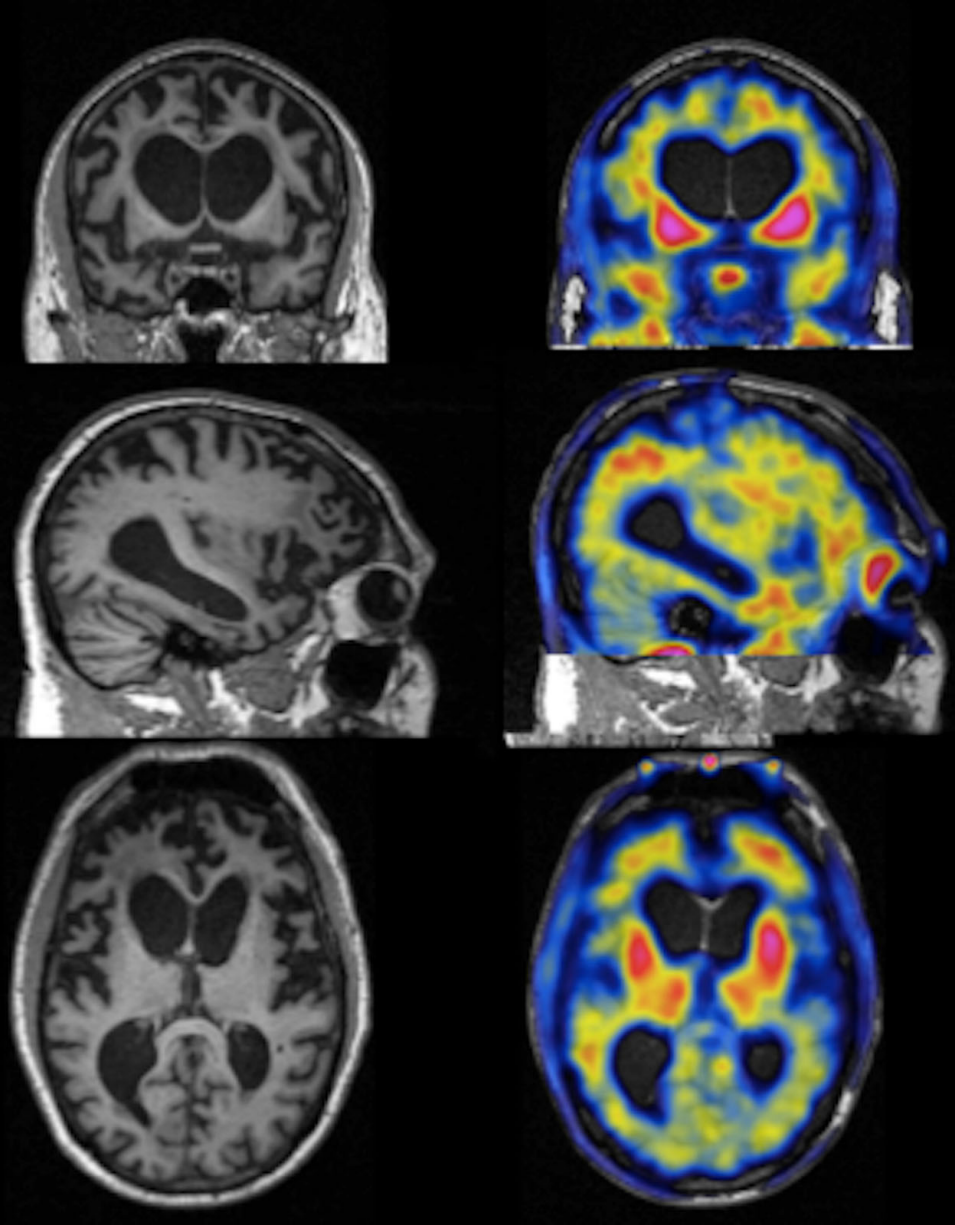
[F18]-T807 PET images from a 56-year-old individual with frontotemporal dementia and the P301L *MAPT* mutation. Coronal (top row), sagittal (middle row) and axial (bottom row) views of prefrontal and anterior temporal atrophy with white matter signal change on MRI (left column) and [F18]-T807 images (right column) showing elevated signal in frontal, anterior temporal and parietal cortex, as well as in basal ganglia, consistent with expected tau inclusions. The PET reference region was the cerebellar grey matter.

A study at the Massachusetts General Hospital has begun to analyse *MAPT* mutation carriers with [F-18]-T807 PET. A 56-year-old man with the P301L mutation has been followed from prodromal FTD to bvFTD associated with an extrapyramidal syndrome. A [F-18]-T807 PET scan obtained 3.5 years from the onset of the behavioural symptoms (Figure 8) demonstrates robust signal in a classic frontotemporal distribution characteristic of inherited tauopathies and with remarkable similarity to the map of pathology described in the MSTD family by Spina and colleagues [108]. Sparing of the occipital cortex contrasts with severe involvement of the anterior and temporal regions of the telencephalon. Although involvement of the basal ganglia is variable in sporadic FTLD-tau, many *MAPT* cases have prominent pathology there. These studies are promising for the further characterization of patients with *MAPT* mutations.

**Figure 9:**
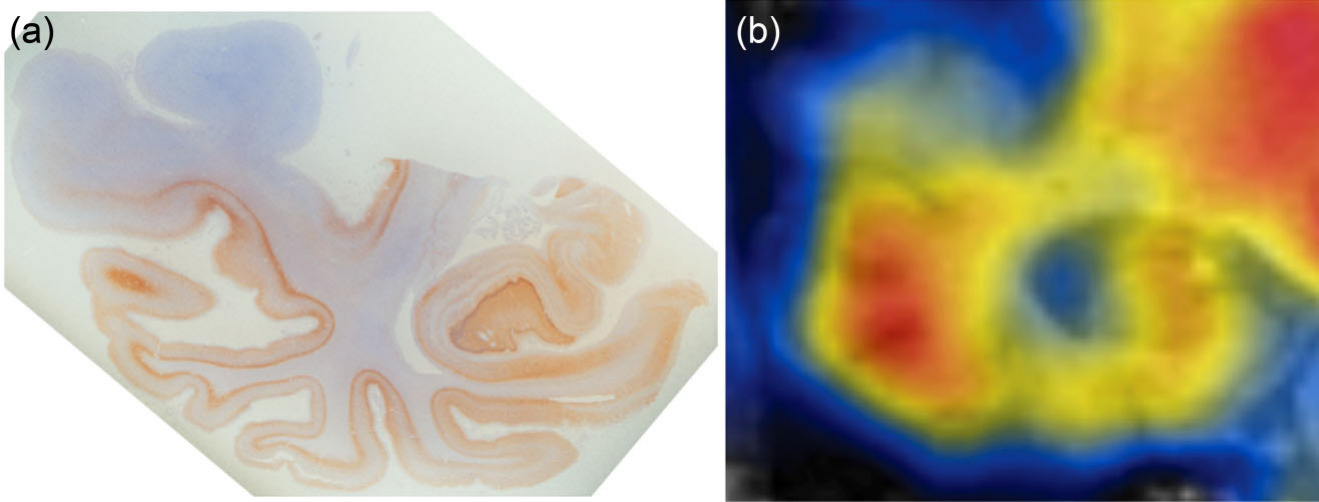
Post-mortem tau immunohistochemistry using AT8 (a) and *in vivo* [F-18]-T807 imaging (b) from two individuals with the P301L *MAPT* mutation. Tau-immunoreactivity is observed in the middle temporal gyrus, inferior temporal gyrus, fusiform gyrus, parahippocampus, entorhinal cortex and hippocampus. The superior temporal gyrus is spared (a). *In vivo* imaging using T807 demonstrates tau binding in the middle temporal gyrus, inferior temporal gyrus, fusiform gyrus, parahippocampus, entorhinal cortex and hippocampus, with no binding in the superior temporal gyrus (b).

Comparative analysis of post-mortem tau immunohistochemistry with *in vivo* [F-18]-T807 PET is essential for understanding the sensitivity of the tracer and the evolution of hyperphosphorylated tau protein deposition. The post-mortem pattern of tau distribution, in the temporal cortex and hippocampus of a 62-year-old patient carrying the P301L *MAPT* mutation and symptomatic for 10 years, is shown in Figure 9. This image is compared with a PET scan obtained *in vivo* using the [F-18]-T807 tracer from the 56-year-old patient carrying the *MAPT* P301L mutation just described. Images obtained from immunohistochemistry and PET imaging reveal tau involvement in the middle temporal gyrus, parahippocampus, entorhinal cortex and hippocampus in both cases, as well as the sparing of the superior temporal gyrus.

*In vivo* tau imaging coupled with neuropathological investigation will improve our understanding of tau spreading in the brain and bring forward knowledge of the large number of disorders characterized by tau deposition [160],[161].

## Clinical features

The onset of FTDP-17 *MAPT* is typically insidious. Individuals with fully developed clinical syndromes usually exhibit at least two of the three cardinal symptoms, which are behavioural and personality disturbances, cognitive impairment and/or motor dysfunction (most often in the form of an extrapyramidal/parkinsonism plus syndrome). Nevertheless, there is substantial heterogeneity. Moreover, clinical variability is seen in individuals with the same *MAPT* mutation, in different families or even within the same family (for details about clinical presentation, see Ghetti *et al*. [17]).

The behavioural and personality abnormalities include disinhibition, apathy, loss of empathy, emotional flatness, impulsive and/or compulsive behaviour, lack of regard for personal hygiene, hyperorality including excessive use of alcohol or other drugs, and in some cases verbal and/or physical aggressiveness. The cognitive symptoms commonly observed in early stages of disease include inattention and executive dysfunction (e.g. difficulty initiating or completing activities or tasks, disorganization, impaired judgment and decision making) with relative preservation of memory, orientation and visuospatial function, thus fulfilling criteria for behavioral variant FTD (bvFTD) [162]. Family members may report memory loss in daily life, but this is often a reflection of the effects of attentional or executive dysfunction on encoding or retrieval. However, some patients with FTDP-17 *MAPT* present with a profound amnestic syndrome [33]. Similarly, the literature contains statements about semantic dementia being a possible clinical phenotype of FTDP-17 *MAPT*, but all cases, except one, also had a behavioural phenotype [163]. A progressive loss of person-specific semantic memory with prominent anomia and right temporal polar atrophy, as well as other characteristics of semantic dementia, was described in an individual with the V363I *MAPT* mutation [119]. Thus, semantic memory in FTDP-17 deserves further investigation.

Progressive nonfluent aphasia may be seen initially [118], but more commonly, an adynamic aphasia syndrome occurs in which the patient speaks very little due to a loss of generative aspects of language. Later, progressive deterioration of memory, orientation and visuospatial function, as well as echolalia, palilalia, and verbal and vocal perseverations, are encountered. Finally, progressive dementia encompassing most cognitive domains develops, and patients often become mute. Motor signs are dominated by parkinsonism, which can be the presenting sign, with some patients being misdiagnosed as having PD or PSP. However, in some families, parkinsonism occurs late or not at all. Parkinsonism associated with FTDP-17 *MAPT* is characterized by symmetrical bradykinesia, postural instability and rigidity affecting axial and appendicular musculature, absence of resting tremor, and poor or no responsiveness to levodopa. Parkinsonism is an early feature of the N279K mutation, and asymmetric resting tremor and levodopa responsiveness have been observed [14]. Other motor disturbances may include dystonia, supranuclear gaze palsy, upper and lower motor neuron dysfunction, myoclonus, postural and action tremor, apraxia of eyelid opening and closing, dysphagia, and dysarthria.

Although essentially no systematic work has been published on genotype–phenotype correlations in FTDP-17 *MAPT*, anecdotal observations suggest that exonic mutations that do not affect the splicing of exon 10 are usually associated with a dementia-predominant phenotype. In contrast, intronic and exonic mutations that affect exon 10 splicing and lead to an overproduction of four-repeat tau tend to be associated with a parkinsonism plus-predominant phenotype.

## Conclusion

This review emphasizes the protean nature of FTD associated with *MAPT* mutations, as well as the need for correlating longitudinal clinical and neuropsychological studies with neuroimaging. Ideally, this research should be carried out both before the onset of symptoms and during the disease in individuals with mutations that differentially affect tau isoforms. These studies, in conjunction with the neuropathological description of tau inclusions, will provide a precise characterization of phenotypic variants and may clarify the anatomical and cellular substrates of each phenotype, as well as the evolution of tau aggregate propagation.
